# Production of bioadsorbent from phosphoric acid pretreated palm kernel shell and coconut shell by two-stage continuous physical activation via N_2_ and air

**DOI:** 10.1098/rsos.180775

**Published:** 2018-12-19

**Authors:** Chuan Li Lee, Paik San H'ng, Md Tahir Paridah, Kit Ling Chin, Umer Rashid, Mariusz Maminski, Wen Ze Go, Raja Ahamd Raja Nazrin, Siti Nurul Asyikin Rosli, Pui San Khoo

**Affiliations:** 1 Institute of Tropical Forestry and Forest Products, Universiti Putra Malaysia, 43400 UPM Serdang, Selangor, Malaysia; 2 Faculty of Forestry, Universiti Putra Malaysia, 43400 UPM Serdang, Selangor, Malaysia; 3 Institute of Advance Technology, Universiti Putra Malaysia, 43400 UPM Serdang, Selangor, Malaysia; 4 Faculty of Wood Technology, Warsaw University of Life Sciences–SGGW, 159 Nowoursynowska Street, 02-787 Warsaw, Poland

**Keywords:** bioadsorbent, two-stage continuous physical activation, palm kernel shell, coconut shell, H_3_PO_4_ pretreatment

## Abstract

In the present study, agricultural biomass—palm kernel shell (PKS) and coconut shell (CS)—was used to produce high porosity bioadsorbent using two-stage continuous physical activation method with different gas carrier (air and N_2_) in each stage. The activation temperature was set constant at 600, 700, 800 or 900°C for both activation stages with the heating rate of 3°C min^−1^. Two parameters, the gas carrier and activation temperature, were determined as the significant factors on the adsorption properties of bioadsorbent. BET, SEM, FTIR, TGA, CHNS/O and ash content were used to elucidate the developed bioadsorbent prepared from PKS and CS and its capacity towards the adsorption of methylene blue and iodine. The novel process of two-stage continuous physical activation method was able to expose mesopores and micropores that were previously covered/clogged in nature, and simultaneously create new pores. The synthesized bioadsorbents showed that the surface area (PKS: 456.47 m^2^ g^−1^, CS: 479.17 m^2^ g^−1^), pore size (PKS: 0.63 nm, CS: 0.62 nm) and pore volume (PKS: 0.13 cm^3^ g^−1^, CS: 0.15 cm^3^ g^−1^) were significantly higher than that of non-treated bioadsorbent. The surface morphology of the raw materials and synthesized bioadsorbent were accessed by SEM. Furthermore, the novel process meets the recent industrial adsorbent requirements such as low activation temperature, high fixed carbon content, high yield, high adsorption properties and high surface area, which are the key factors for large-scale production of bioadsorbent and its usage.

## 1. Introduction

Used since the ancient times by Egyptians and Indians, activated carbon is a unique and versatile adsorbent to eliminate undesirable odour, taste, dyes, heavy metals and organic substances. In contemporary times, activated carbon has been extensively used by the chemical industries for removal, separation and preconcentration of both metallic and organic species from water and wastewater [1]. Today, coal-based activated carbon is the most prevalent adsorbent used; however, it is expensive and non-sustainable. This has led to a search for low-cost, easily available materials as alternative adsorbent materials.

Conversion of lignocellulosic biomass to high-efficiency bioadsorbent would boost its economic value, develop economical adsorbent materials and inspire diversion of the waste away from landfill and open burning. In Malaysia, enormous amounts of palm kernel shell (PKS) and coconut shell (CS) are still underused. Chiefly, both of this lignocellulosic biomass has the aptitude to be used as inexpensive adsorbents as it is not only an underused resource but practically at hand and sustainable resource [2,3]. Thence, the application of these inexpensive lignocellulosic biomass wastes as the carbon precursor through thermochemical conversion is very promising [4]. The adsorbent can present specific physico-chemical characteristics (i.e. surface area, porosity, pore-size distribution and surface functional groups) depending on the precursor and the method used in its preparation [5,6]. Industrially, cheap carbonaceous precursors with high carbon and low inorganic (i.e. ash) contents are preferred as raw material for the production of adsorbent [6]. High mineral (ash) content in agricultural biomass such as PKS and CS creates a significantly different challenge for preparing high-quality activated carbon. Currently, carbon from this high-ash agricultural biomass generated in thermochemical conversion has only been used as a soil amendment or low-grade fuel [7,8]; this inherently limits the market demand for this type of carbon. Ash leaching method with phosphoric acid prior to physical activation has been proven to remove a huge fraction of ash-forming elements in biomass and expose the mesopores of the carbon produced [9,10]. However, for high-ash agricultural biomass such as PKS and CS, the pores are majorly clogged by ash components such as silica or silicate derivatives, which are difficult to remove using the leaching method. To unclog the existing mesopores and create micropores, the biomass has to undergo further treatment, physical activation [11].

Physical activation using only nitrogen as the carrier gas to produce adsorbent has been carried out by many researchers [12–20]. Nitrogen is a small-scale molecule that can penetrate into narrow, smaller and deeper inside of particles to create micropores [21,22]. However, using nitrogen gas as carrier gas produced a very small surface area and enormous amount of ash, leading to inhibition of the activation especially on high-ash biomass due to the lack of open mesopores structure in the raw material itself [23]. While, using air as the carrier gas, it primitively modifies the bioadsorbent adsorptive affinity for organic compounds by opening up pores and introducing strong hydrogen bonding sites for ionizable compounds [11]. Thermal air activation of biomass can modify the pore size distribution and connectivity, and promote the oxygen functionality [11,24]. High-ash biomass might develop from this air activation as it may help open up pores that are clogged by major ash components such as silica or silicate derivatives and create the mesopores structure on the carbon produced. However, when air was present in activation process with high temperature and prolong period, the biomass might burn and cause the char yield to decrease [25].

Physical activation of high-ash biomass via air and N_2_ help in creating different types of pores: mesopores and micropores, respectively. Thus, two-stage continuous physical activation was suggested in this study. Incorporating two-stage continuous physical activation via air and N_2_ on phosphoric acid treated high-ash biomass will certainly increase the quality of the bioadsorbent produced. No similar work has been reported so far for bioadsorbent produced via two-step continuous physical activation with nitrogen and air as carrier gas which suggests a need for the present work. This study suggests that it is feasible to control the sequence of the gas and activation temperature by means of the experimental parameters. The phosphoric acid pretreated biomass was physically activated in two stages using air or N_2_ as the gas carrier in each stage (Air > N_2_ or N_2_ > Air). The variations in properties of the bioadsorbent produced were analysed. One-stage physical activation using only N_2_ or only air as the gas carrier was also conducted to create a comparison between one-stage and two-stage continuous physical activation of phosphoric acid treated PKS and CS. As a novelty, we include the significant difference in bioadsorbent characteristics of bioadsorbent prepared via one-stage and two-stage physical activation. Analysis of variance (ANOVA) was used to test the main effects of the suggested physical activation method (one-stage physical activation: Air or N_2_, and two-stage continuous physical activation: Air > N_2_ or N_2_ > Air) and activation temperature on the characteristics of adsorbent derived from phosphoric acid treated CS and PKS. Furthermore, the determination of the BET (Brunauer–Emmett–Teller) internal pore surface area and volume, structural analysis of the carbon crystals and pore bodies from the scanning electron microscope (SEM), chemical composition through CHNS/O and ash content while the surface chemical characteristics determined by Fourier transform infrared (FTIR) and thermal behaviour through thermogravimetric analysis (TGA) were also taken as responses. Thus, this study not only provides the adsorption capabilities of biomass from the suggested physical activation methods but also insight into the design and performance of bioadsorbent from high-ash biomass with high adsorption value.

## 2. Material and methods

### 2.1. Preparation of samples prior to physical activation (H_3_PO_4_ pretreatment)

CS and PKS were collected, cleaned and dried in an oven at 105°C for 48 h. The dried raw materials were then crushed and sieved to 2–5 mm size range. Specified mass of the dried PKS and CS were impregnated with 30% phosphoric acid (H_3_PO_4_) with impregnation mass ratio of 1 : 1 at 80°C for 2 h. The pretreated particles were filtered and washed with distilled water. The washed pretreated particles were dried at 105°C for 48 h.

### 2.2. Physical activation process

The process of physical activation was performed in a horizontal laboratory tube furnace. For one-stage physical activation, the H_3_PO_4_ pretreated samples were activated for 75 and 105 min with air or N_2_ gaseous (flow rate = 100 ml min^−1^) at temperature 600, 700, 800 and 900°C, respectively. After, the one-stage physical activation, the activated samples were kept in desiccators for further analysis.

For two-stage continuous physical activation, the H_3_PO_4_ pretreated samples were activated continuously in two stages of different gas carrier: air and N_2_. The activation temperature was set constant at 600, 700, 800 or 900°C for both activation stages with the heating rate of 3°C min^−1^. For ‘N_2_ > Air’ activation, the pretreated samples were activated in N_2_ (flow rate = 100 ml min^−1^) for 75 min before undergoing air activation (flow rate = 100 ml min^−1^) for 30 min. While, for ‘Air > N_2_’ activation, H_3_PO_4_ pretreated samples were activated with air (flow rate = 100 ml min^−1^) for 75 min and followed by N_2_ activation (flow rate = 100 ml min^−1^) for 30 min. Samples were then cooled to room temperature by flowing N_2_ through the activated samples (flow rate = 100 ml min^−1^). After, the two-stage continuous physical activation, the activated samples were kept in desiccators for further analysis.

### 2.3. Characterization

The detail of the standard method testing process and calculation for the performance of bioadsorbent included BET surface area, specific surface area characteristics and iodine adsorption number was mentioned in our previous study [26]. In this study, methylene blue adsorption number was determined on the basis of Standard Method JIS K 1470–1991 [27,28]. The surface chemical characteristics of CS and PKS were determined by FTIR in the range of 700–4000 cm^−1^. TGA was done using Perkin Elmer, USA, equipment. The experiments were performed in an inert atmosphere with a continuous flow of argon at the rate of 50 ml min^−1^ and heated at a heating rate of 10°C min^−1^. About 10 mg of samples was placed on a balance located in the furnace tube and heat was applied over the temperature range from 0 to 1000°C. In addition, the ultimate analysis of the bioadsorbent was performed in CHNS/O analyser (Perkin Elmer PE2400 Series *ΙΙ*). TAPPI standard method, T211 om-85 was used to determine the ash content. The oven-dried sample (2 g) was burned (dry oxidation) in a muffle furnace at 575 ± 25°C for 4 h. This standard test method was used to determine the volume of ash remaining after dry oxidation of the sample [29].

### 2.4. Data analysis

Statistical package for the social science (SPSS) was applied to evaluate the data of adsorption properties of bioadsorbent for ANOVA at 95% confident level (*p* ≤ 0.05). Tukey–Kramer multiple comparison test was applied to analyse the differences of the treatment effects when significance was observed. The effects were considered not statistically significant when the *p*-value was higher than 0.05 at the 95% confidence level.

## 3. Results and discussion

### 3.1. Adsorption properties of bioadsorbent

The adsorption properties of bioadsorbent derived from H_3_PO_4_ pretreated CS and PKS were compiled in table 1. For all the models, the ANOVA analysis showed *p*-value was less than 0.01 for the parameters, physical activation, activation temperature and gas carrier × activation temperature, signifying that the models were stiffly significant at the 99% confidence level for CS and PKS bioadsorbent. We may observe that there is an increase in the adsorption properties of the pretreated bioadsorbent as compared to the non-treated bioadsorbent. This could potentially be due to the removal of some components, e.g. tar-like matter, and phosphoric acid deposited in pores, which occurred better at higher activation temperature, leading to the development of micropores and mesopores [30]. Table 1 illustrates the effect of physical activation and activation temperature on adsorption of methylene blue using PKS and CS bioadsorbent. Activation temperature influenced the adsorption properties in two competitive ways. First and foremost, it was able to promote the diffusion rate of adsorbate molecules across the external boundary layer and internal pores of the adsorbent will enhance the adsorption rate. Furthermore, it can attenuate the force between adsorbent and adsorbate, improving the desorption rate [31]. The adsorption of methylene blue on both the bioadsorbents decreased significantly when the activation temperature went up to 900°C. The decrease of adsorption rate might be due to the limited quantities of available active sites on the surface of the bioadsorbent [32]. The quantity of active sites accessible for methylene blue adsorption rises once the porosity of the adsorbent increases [33]. Hence the result showed that high activation temperature will cause the decreased of porosity in bioadsorbent. Table 1 demonstrates that the air activated bioadsorbent obtained the highest methylene blue adsorption. This result verifies that the air activation promotes mesopore in bioadsorbent. Comparatively, bioadsorbents undergoing two-stage continuous physical activation achieved higher methylene blue adsorption than one-stage physical activation. Oxygen from air acted as a catalyst to enlarge or create mesopores in carbonaceous materials. The highest methylene blue adsorption (208.40 mg g^−1^) for CS adsorbent was obtained when the CS was activated at 800°C in Air > N_2_ activation. On the other hand, PKS sample activated at 700°C under Air > N_2_ activation achieved the highest methylene blue adsorption (253.26 mg g^−1^). Eventually, Air > N_2_ physical activation delivered bioadsorbent with a highly mesoporous surface.

**Table 1.** ANOVA for methylene blue and iodine adsorption of CS and PKS bioadsorbent (control—bioadsorbent without H_3_PO_4_ pretreatment). Note: means followed by the same letter in the same column are not significantly different at *p* ≤ 0.05 according to Tukey multiple comparison test.

physical activation			adsorption properties			
			methylene blue adsorption (mg g^−1^)		iodine adsorption (mg g^−1^)	
time (min)	temperature (°C)	gas carrier	CS	PKS	CS	PKS
control (non-treated bioadsorbent)						
raw material			1.79	0.77	1.71	1.28
75	700	Air	104.58	86.13	305.90	286.35
75	700	N_2_	81.51	75.36	314.28	291.94
105	700	Air	64.60	66.90	227.71	202.58
105	700	N_2_	61.52	63.06	233.30	208.17
one-stage physical activation						
75	600	Air	71.00_f_	78.69_e_	277.05_f_	291.01_b_
75	700	Air	149.96_a_	91.25_a_	319.86_b_	299.39_b_
75	800	Air	103.30_c_	82.28_d_	291.94_d_	271.46_c_
75	900	Air	81.26_d_	70.75_f_	280.77_e_	211.89_e_
75	600	N_2_	74.59_e_	64.08_g_	264.01_g_	234.23_d_
75	700	N_2_	107.66_b_	88.18_b_	328.25_a_	305.90_b_
75	800	N_2_	101.25_c_	85.36_c_	325.45_a_	329.17_a_
75	900	N_2_	61.52_g_	83.05_d_	297.52_c_	291.94_b_
*p*-value			<0.001	<0.001	<0.001	<0.001
105	600	Air	81.51_e_	87.92_b_	210.03_e_	203.53_e_
105	700	Air	101.76_a_	93.05_a_	250.05_c_	230.50_b_
105	800	Air	94.59_c_	79.21_e_	185.83_g_	205.37_d_
105	900	Air	77.67_f_	67.93_h_	177.45_h_	147.65_h_
105	600	N_2_	76.13_g_	70.75_g_	202.58_f_	200.72_f_
105	700	N_2_	95.36_b_	84.85_c_	277.05_a_	247.26_a_
105	800	N_2_	87.67_d_	82.28_d_	266.81_b_	213.75_c_
105	900	N_2_	58.19_h_	72.29_f_	214.68_d_	177.45_g_
*p*-value			<0.001	<0.001	<0.001	<0.001
two-stage continuous physical activation						
105	600	Air > N_2_	102.79_g_	245.31_b_	384.09_f_	406.43_d_
105	700	Air > N_2_	140.98_c_	253.26_a_	416.67_e_	431.56_c_
105	800	Air > N_2_	208.40_a_	158.16_d_	439.01_d_	329.17_e_
105	900	Air > N_2_	105.35_f_	85.36_h_	327.31_h_	310.56_f_
105	600	N_2_ > Air	133.29_e_	132.27_e_	525.57_a_	501.37_a_
105	700	N_2_ > Air	164.57_b_	203.53_c_	504.17_b_	481.83_b_
105	800	N_2_ > Air	139.45_c_	95.36_f_	442.73_c_	438.08_c_
105	900	N_2_ > Air	136.37_d_	92.28_g_	347.79_g_	339.41_e_
*p*-value			<0.001	<0.001	<0.001	<0.001

Iodine adsorption is the most fundamental parameter used to define and characterize the performance of adsorbent. Iodine adsorption was used as an approximation of the surface area of an adsorbent as well as a measure of micropores [34]. Besides, adsorbents with a high iodine adsorption number perform better in eliminating small sized contaminants in the field [35]. Table 1 reveals that both bioadsorbents obtained the lowest iodine adsorption via air activation, which may be caused by the high reactivity during air activation. The finding of Kyritsis [36] illustrated that oxygen will react at the entrances of the pores but does not penetrate into the narrow pores. Some researchers had reported that oxidation will partially destruct the micropore walls and, thus, generate low surface area on the adsorbent [37]. Moreover, the process of dehydrogenation occurred under N_2_ activation but not under air activation due to the instability of the residue in the air [38]. The dehydrogenation properties will restrict the formation of tar and diminish the production of other volatile products, resulting in changeable aromatization of the carbon skeleton by the creation of a porous structure and surface area [39]. Under the circumstances, bioadsorbent prepared via N_2_ activation achieved higher iodine adsorption when compared with air activation. Table 1 shows that the iodine adsorption for both bioadsorbents under two-stage continuous physical activation is higher than one-stage physical activation. After performing an ANOVA analysis on two-stage continuous physical activation, the result defined that the highest iodine adsorption was achieved when the lignocellulosic biomass was propelled by the N_2_ > Air activation. The highest iodine number (525.57 and 501.37 mg g^−1^) was recorded in the CS and PKS bioadsorbent produced at 600°C under N_2_ > Air activation. Small pores are adept at adsorbing iodine molecules, and, thus, the quantity of iodine molecules adsorbed is often considered an indicator of the number of micropores in adsorbent [40–43]. The enhancement of iodine adsorption for both bioadsorbents via N_2_ > Air activation indicated the increase of micropores on the bioadsorbent in this study.

Besides, it is proposed that H_3_PO_4_ impregnation not only promotes the pyrolytic decomposition of raw material but also leads to the formation of cross-linked structure [44]. Aside from affecting the development of the pores, particularly the size, H_3_PO_4_ pretreatment also affects the resulting adsorption properties. Experimental results show that the amount of adsorption capability increased dramatically with the H_3_PO_4_ pretreatment. Furthermore, the reaction time of the physical activation has a significant effect on the development of the carbon's porous networks. The reaction time should just be enough to eliminate all the moisture and most of the volatile components in the precursor to cause pores to develop [26]. Ironically, bioadsorbent treated for a shorter time reached a higher adsorptive capacity than bioadsorbent activated for a longer time. This may be partly due to the prolonged reaction time resulting in structural deformation, hence less surface area for adsorption [45]. For this reason, the bioadsorbent physically activated at 105 min achieved a lower adsorption than bioadsorbent prepared in 75 min.

### 3.2. Pore structure and surface area characteristics (BET surface area)

Evaluation of the surface characteristics and pore structures in this study was performed only on the non-treated and pretreated bioadsorbent that obtained the highest adsorption properties from one-stage physical activation and two-stage continuous physical activation.

The properties of the surface and porosity of adsorbent rely on the raw material, parameters of the thermal reaction (temperature and time) and physical activation (surrounding gas composition) [11]. Porosity in carbon materials primitively develops during physical activation due to irregular spacing between aromatic sheets. In spite of that, the pores are consequently clogged up as a result of the deposition of tarry materials [46]. With reference to table 2, the pretreated bioadsorbent achieved a higher surface area than the non-treated bioadsorbent. The high value of BET surface area in pretreated bioadsorbent is indicative of highly developed pore network within the carbon. The pretreated bioadsorbent also has smaller pore size than the non-treated bioadsorbent. The enlargement of surface area for both pretreated bioadsorbents may be accomplished via the creation of new micropores as propounded by the growth in the micropores surface area and pore volume. H_3_PO_4_ is the dehydrating agent that penetrates deep into the structure of the carbon causing tiny pores to develop [47]. Simultaneously, the bioadsorbents derived from the CS and PKS by H_3_PO_4_ pretreatment under appropriate process conditions have higher proportions of microporous and mesoporous with a high surface area. Apart from that, a longer physical activation promoted reduction in both surface area and total pore volume, presumably due to the collapse of smaller pores, as inferred from the decrease in micropore volume [23]. As shown in table 2, the BET surface area of the bioadsorbent prepared by H_3_PO_4_ for 105 min is lower compared to the bioadsorbent prepared under 75 min. It is also lower in terms of percentage of micropore to the BET surface area of the sample. Generally, further increase in the reaction time resulted in the decrease of the micropore distribution, similar to that of BET surface area.

**Table 2.** The surface area and pore size characterization of the prepared bioadsorbent (control—bioadsorbent without H_3_PO_4_ pretreatment).

physical activation			pore structure and surface area characteristics of bioadsorbent					
			BET surface area (m^2^ g^−1^)		pore size (nm)		micropore volume (cm^3^ g^−1^)	
time (min)	gas carrier	temperature (°C)	CS	PKS	CS	PKS	CS	PKS
control (non-treated bioadsorbent)								
raw material			0.84	0.79	6.30	7.21	0.0013	0.0014
75	N_2_	700	91.39	69.42	45.82	82.06	0.03	0.01
one-stage physical activation								
75	Air	700	124.32	99.19	47.13	36.88	0.01	0.0004
75	N_2_	700	147.91	114.55	6.58	6.27	0.06	0.06
105	Air	700	87.75	82.27	73.87	78.43	0.04	0.03
105	N_2_	700	114.55	98.09	7.13	7.55	0.04	0.04
two-stage continuous physical activation								
105	Air > N_2_	700	—	169.76	—	6.07	—	0.06
105	Air > N_2_	800	179.61	—	7.99	—	0.05	—
105	N_2_ > Air	600	479.17	456.47	0.62	0.63	0.15	0.13

**Figure 1.**
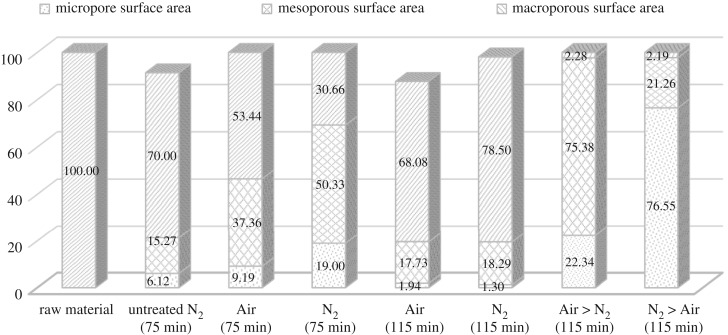
Porosity ratio of CS bioadsorbent.

**Figure 2.**
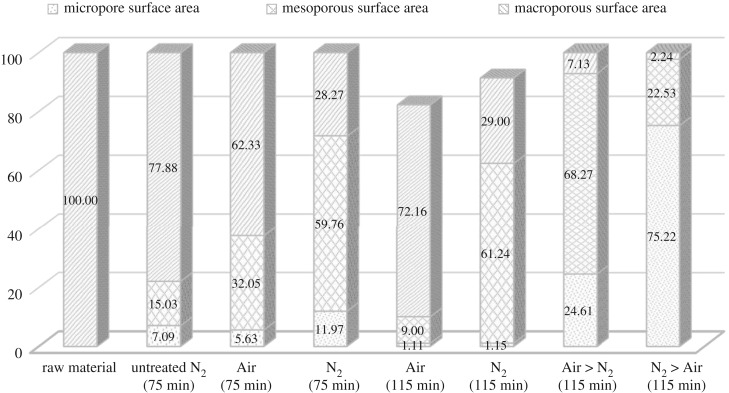
Porosity ratio of PKS bioadsorbent.

In this study, two-stage continuous physical activation is attempted for the purpose of clearing off disorganized carbon and opening up clogged pores, and simultaneously creating new pores by selectively burning off the carbon atoms. In conformity with table 2, figures 1 and 2, two-stage continuous physical activation leads to a considerable increase in both the BET surface area and porosity including micropore surface area and micropore volume as compared to single activation. Figures 1 and 2 also depict the porosity ratio distribution of the produced bioadsorbent. The micropore porosity ratio for bioadsorbents prepared under two-stage continuous physical activation was higher than one-stage physical activation. The porosity ratio results declared that bioadsorbent prepared via Air > N_2_ activation produced the higher mesoporous structure. This result directly related to the methylene blue adsorption results, as bioadsorbent prepared via Air > N_2_ activation obtained the highest methylene blue adsorption (table 1). Meanwhile, a significant decrease in pore size for samples prepared under N_2_ > Air activation implied that this activation method promotes the formation of narrower porosity (eight times smaller than the sample prepared by Air > N_2_ activation). Table 2 also shows the highest micropore volume (0.15 and 0.13 cm^3^ g^−1^ for CS and PKS bioadsorbent, respectively) resulted when both bioadsorbents were activated under N_2_ > Air activation. The results denoted that N_2_ > Air activation elicits an increase in pore volume and reduction of pore size, resulting in high surface area for both bioadsorbents. The highest BET surface area was obtained from bioadsorbent prepared under N_2_ > Air activation with 482.24 and 456.47 mg g^−1^ for CS and PKS, respectively. This may be explained by the oxygen groups probably being fixed at the most active sites at the entrance of the micropores during the pre-oxidation stage for Air > N_2_ activation. Therefore, the access of nitrogen to these micropores becomes difficult [48], and as consequence, adding N_2_ activation after Air activation was not able to induce many pores, thus, the specific surface area was not greatly increased. On the contrary, N_2_ > Air activation promotes the bioadsorbent with higher microporous structure. CS and PKS bioadsorbent contained 76.55% and 75.22%, respectively, of micropore distribution when prepared under N_2_ > Air activation. The observed increases in micropore distribution were primarily ascribed to the thermal decomposition of surface functional groups from the inside of pores upon pre-heat treatment with nitrogen [49–51]. This preferentially leads to micropore formation instead of pore enlargement which explains why the pore size of the sample via N_2_ > Air activation was approximately 10 times smaller than the other samples. This is well visualized by SEM image. The SEM image of bioadsorbent prepared via N_2_ > Air activation (figure 5*o,*5*p* shows that the microscopic shape of bioadsorbent is an agglomeration of sub-micrometre particles. The enhancement of the formation of micropore surface area by N_2_ > Air activation is favourable to promote higher adsorption for the bioadsorbent. The high micropore distribution translates the suitability of the activation method used in this study. Thus, N_2_ > Air activation method was found to produce the highest quality of bioadsorbent among the activation methods used in this study. CS and PKS bioadsorbents prepared under N_2_ > Air activation acquire a high potential in activated carbon application with its comparable high surface area.

Table 3 shows the characteristics of bioadsorbents produced in this work and also from other recent studies by other researchers. It shows the results of the surface area characteristic of the bioadsorbent made from different precursors and condition of activation. Among them, the bioadsorbent produced in this study apparently possesses the best quality in terms of its porosity characteristics. CS and PKS prepared via two-stage continuous physical activation at 600°C are preferable because they possess higher BET surface areas. In other words, they have higher adsorption sites for molecules to attach onto the surface of the carbon.

**Table 3.** Comparison of preparation and characteristics of bioadsorbent from this work with other studies.

references	biomass	activation condition	BET value (m^2^ g^−1^)
present work	CS	two-stage continuous physical activation	479.17
present work	PKS	two-stage continuous physical activation	456.47
[52]	PKS	KOH at 800°C for 45 min	119.3
[53]	empty fruit bunch	(700°C) for 2 h under N_2_ flow (150 cm^3^ min^−1^)	231.52
[53]	oil palm mesocarp fibre	(700°C) for 2 hr under N^2^ flow (150 cm^3^ min^−1^)	239.50
[54]	cotton seed shell	sulfuric acid	124.35
[55]	corn husk and sugarcane bagasse	carbonized with temperature 500°C for 2 h in N_2_ and continued activate in air atmosphere for 40 min	256
[56]	date stone	physical (N_2_ and steam) at 700°C for 60 min	322

### 3.3. N_2_ adsorption

Evaluation of the surface characteristics and pore structures in this study was performed only on the non-treated and pretreated bioadsorbent that obtained the highest adsorption properties from one-stage physical activation and two-stage continuous physical activation.

**Figure 3.**
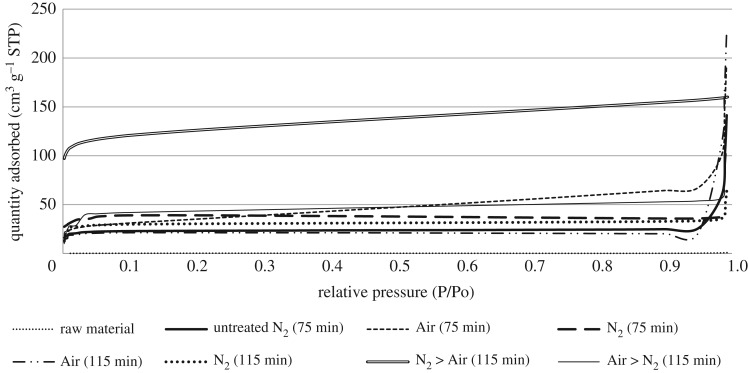
N_2_-Adsorption isotherm for the sample at 77 K for CS bioadsorbent.

**Figure 4.**
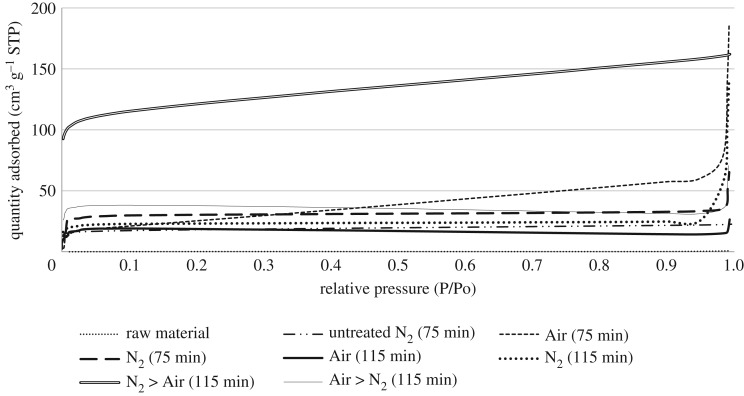
N_2_-Adsorption isotherm for the sample at 77 K for PKS bioadsorbent.

Nitrogen adsorption is the standard channel to evaluate the porosity of carbonaceous adsorbent. The adsorption isotherm provides the information source for the porous structure of the adsorbent, heat of adsorption, physico-chemical characteristics and others. Figures 3 and 4 established the N_2_-adsorption isotherm acquired from the raw material, non-treated bioadsorbent and bioadsorbent sample prepared from CS and PKS under the optimum conditions (bioadsorbent with highest adsorption properties from one-stage physical activation and two-stage continuous physical activation). Most isotherms have been shown to conform to one of the five types of International Union of Pure and Applied Chemistry (IUPAC) classification. Both precursors and non-treated bioadsorbent conform to type II isotherm, where B points out the zone where the monolayer becomes filled. The inflection point of the isotherm usually occurs near the completion of the first adsorbed monolayer and with increasing relative pressure, second and higher layers are completed until at saturation the number of adsorbed layers becomes infinite. This indicated that both precursors were non-porous or whole externally. While both high-ash agricultural biomass that had undergone one-stage physical activation (only Air and only N_2_) and Air > N_2_ two-stage continuous physical activation conform to type IV isotherm. This indicated a significant microporous and mesoporous network system present in both bioadsorbents. From the result obtained, both bioadsorbents prepared using N_2_ > Air activation conform to type I isotherm, disclosing that the bioadsorbents are given by microporous solids having relatively small external surfaces such as activated carbons, molecular sieve zeolites and certain porous oxides.

### 3.4. Scanning electron micrograph

Evaluation of the surface characteristics and pore structures in this study was performed only on the non-treated and pretreated bioadsorbent that obtained the highest adsorption properties from one-stage physical activation and two-stage continuous physical activation.

**Figure 5.**
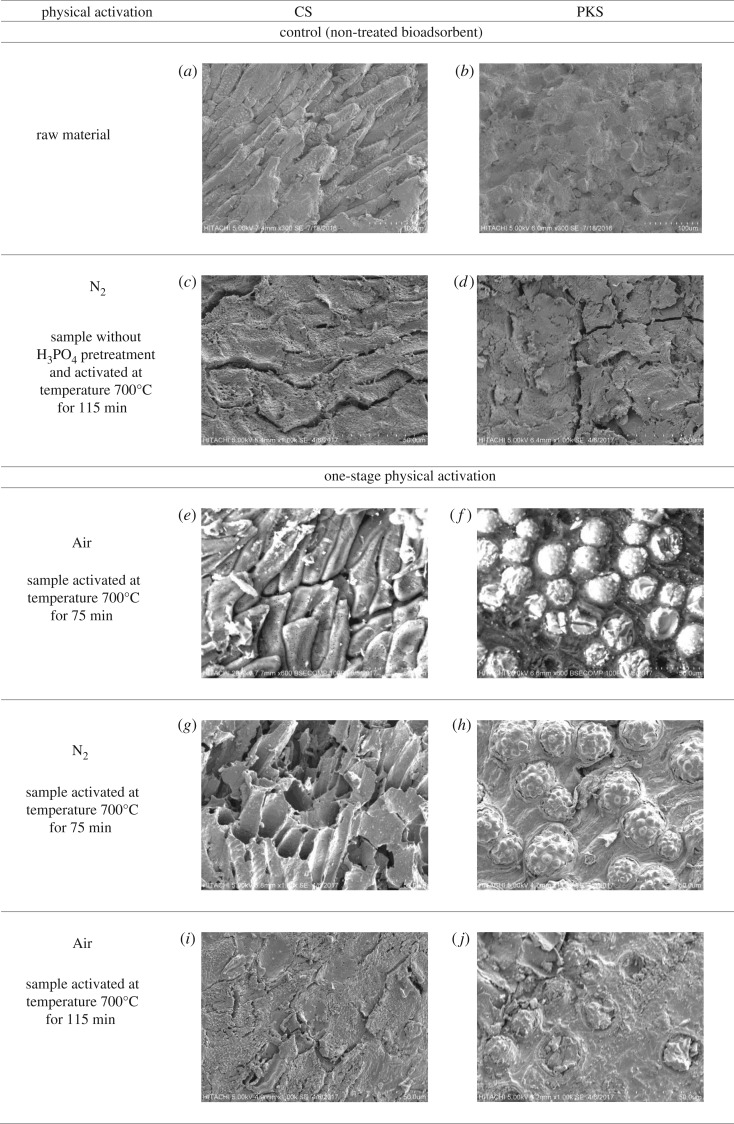

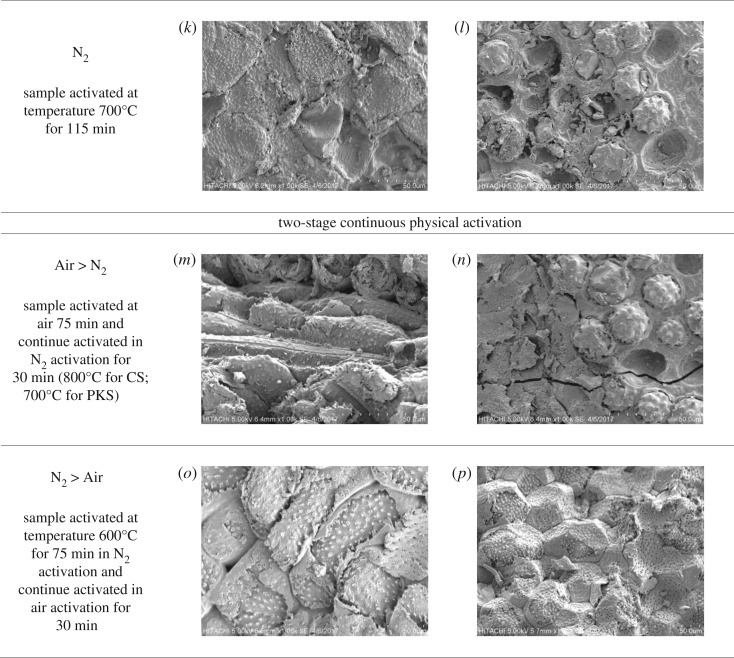
(*a*–*p*) Scanning electron micrograph of bioadsorbent activated under different physical activation in low magnification (50 µm).

Figure 5*a,b* presents the micrographs for CS and PKS employed as the raw material for the preparation of bioadsorbent. The surface of the raw material was dense and planar without any cracks and crevices. This would account for its poor or negligible BET surface area (table 2). As shown in figure 5*a,b*, we noticed that the surface of raw material CS shows holes that were spaced out on the surface with smooth edges, whereas raw material PKS was smooth without any ridges or pores. Most of the pores of the CS were closed up and the pores for PKS were not visible at the magnification of the SEM. After the N_2_ physical activation, the surface morphology showed cracks but no visible pore development occurred on both non-treated bioadsorbents (figure 5*c,d*). Phosphoric acid pretreatment preserved better starting structure of CS and PKS bioadsorbent. This phenomenon is in relation to the pore structure and surface area characteristics surface morphology of the pretreated bioadsorbent (table 2). Despite that, the cellular structure of CS and PKS was noticeable after chemical pretreatment and physical activation. Large amounts of orderly pores are developing on the pretreated CS and PKS bioadsorbent surface with broken edges (figure 5*e,f*). This was attributed to a small amount of impurities such as tar that may cause the pore to clog up and inhibit good pore structure development [52]. Thus, activation stage (H_3_PO_4_ pretreatment) was able to produce an extensive external surface with high surface area on the pretreated bioadsorbent. In general, the choice of the reaction time for the activation depends on the desired porous structure distributions. Except for a lot of ‘nodule-like’ structures which can be clearly seen in the external surface of the pretreated bioadsorbent which was prepared for 105 min (figure 5*i*–*l*), no significant change is observed in the exterior surface structure compared to the bioadsorbent prepared for 75 min, thereby indicating that the gasification occurs mainly in the interior of the particles and presumably is channelled out to the ‘nodule-like’ structures.

According to figure 5*e,f*, when both lignocellulosic biomasses are prepared under air activation, we noticed that the surface becomes coarser/rougher or more plicated, presumably resulted from the loss of char mass under oxygen attack. Some dirt-covered/impurities and unclear pores were present on the surface of the CS bioadsorbent (figure 5*e*). Such rudimentary porous structure and deposition of tarry substances in the interstices of the sample was caused by the incomplete volatile release [6,57–59]. In contrast, the surface morphology of PKS (figure 5*f*) with large pores was covered with silica. Oxygen from the high reactivity of air activation reacted at the entrances of the pores and does not penetrate into the narrow pores was stated by Savova *et al.* [60]. Henceforth, this may clarify that bioadsorbent prepared with air activation produced an oxidized carbon with the low BET surface area and low micropore porosity ratio 9.19 and 5.16% for CS and PKS bioadsorbent. Further, the bioadsorbents induced by air consisted of the surface area with a predominantly macroporous structure with the pore size of 6.58 and 6.27 nm for CS and PKS bioadsorbent, respectively (table 2). Correspondingly, some researchers proved that using nitrogen gas as carrier gas produced a very small surface area [16,23]. Better information may be obtained from figure 5*g*; carbon skeleton structure appeared on the surface of CS under N_2_ activation. The smooth surface area with absence of pores urged the low BET surface area for CS bioadsorbent prepared under N_2_ activation. Besides, the micrographs (figure 5*d*) manifested that the surface condition of the PKS bioadsorbent was primitively hard and rough, and the pores were clogged by the silica bodies. Based on the study of Razali *et al.* [61] silica acted to protect the biomass structure and enhance its mechanical strength. Figure 5*h* demonstrated the silica still present on the bioadsorbent activated with N_2_ activation. The presence of the silica on the surface of bioadsorbent will produce limited surface area, suggesting an inhibition of the reaction. The limited surface area may be caused by the low micropore volume; as demonstrated in table 3, the micropore volumes for CS and PKS bioadsorbents are only 0.06 and 0.04 cm^3^ g^−1^, respectively.

Additional physical activation stage via N_2_ after air activation (Air > N_2_) for 75 min had reduced the silica compounds that naturally attached to the surface of CS and PKS (figure 5*m,n*). The rough appearance was observed at CS adsorbent produced under Air > N_2_ activation. The formation of longitudinal cells in the transverse section shows the fragility and lightness of the CS bioadsorbent. Likewise, the open pores appearing on the CS bioadsorbent surface can be due to the reduction of volatile species. On the other hand, occasional cracking occurred on the surface area of PKS activated under Air > N_2_ activation method. The cracking was probably due to the drastic combustion when oxygen was present during the air activation. The clogged-up pore and drastic combustion had inclined the BET surface value (179.61 and 169.76 mg g^−1^ for CS and PKS) for bioadsorbents prepared under Air > N_2_ activation. Additional stage of air activation after N_2_ activation (N_2_ > Air) increased the development of pores structure on the bioadsorbent (figure 5*o,p*). The well-developed porous structure was due to the significant reduction of the silica compounds attached to the surface of CS and PKS, which open up more reactive surfaces for the activation process. When the sample was prepared under N_2_ > Air activation, the CS surface structures (figure 5*o*) have burnt-out pore with tunnel. The unclogged pore contributed high BET surface area with high micropore porosity ratio (76.55%) for CS bioadsorbent stimulated under N_2_ > Air activation. At the same time, the morphology of PKS attests substantial changes provoked by N_2_ > Air activation. Figure 5*p* clearly attested the porous nature with a predominant microporous character (pore size for PKS bioadsorbent is 0.63 nm) responsible for the developing of high surface area and high iodine adsorption. In addition, figure 5*o,p* also proved that N_2_ > Air activation managed to unclog the pores deposited with tarry material, silica or silicate derivatives during the process of physical activation. The surface morphology in figure 5*o,p* shows favourable characteristics for high adsorption capability, elucidating that N_2_ > Air two-stage continuous physical activation is effective in creating well-developed pores for CS and PKS bioadsorbents.

Overall, it was observed that bioadsorbent generated under one-stage physical activation failed to create sufficient porosities, caused by the deficient decomposition of organic constituents existing in the carbonaceous precursors. Thus, the pores were significantly clogged by the residues of products, leading to a decreased surface area with less porosity. N_2_ > Air activation prompted the bioadsorbent with the surface area consisting of abundant micropores, instigating the high BET surface area for both bioadsorbents. The SEM results have displayed that N_2_ > Air activation created new ultra-micropores and enlarged existing pores to generate new supermicropores and mesopores. Besides, N_2_ > Air activation would have made previously closed ultra-micropores accessible. Lignocellulosic biomass generated with N_2_ > Air activation thereupon was suggested as it was able to reduce the formation of silica, which blocked the pores and constrained the development of pores structures.

### 3.5. CHNS/O and ash content

Evaluation of the element data in this study was performed only on the non-treated and pretreated bioadsorbent that obtained the highest adsorption properties from one-stage physical activation and two-stage continuous physical activation.

The physico-chemical characteristics and potential uses of adsorbent are greatly influenced by the biomass feedstock, feedstock pretreatments, carbonization method and activating conditions, such as temperature, gas type and reaction time [62]. Table 4 presents the contents of carbon, hydrogen, nitrogen, oxygen and ash content of the bioadsorbent. Compared with the non-treated bioadsorbent, H_3_PO_4_ pretreated bioadsorbent displayed a very distinctive chemical composition. The carbon content was much higher, whereas its contents of hydrogen, nitrogen, sulfur and ash, were lower. This distinctive chemical composition suggests that the H_3_PO_4_ pretreated bioadsorbent might have its unique structure, which would considerably affect its adsorption behaviour. For instance, the high-ash content could render the bioadsorbent less polar and give rise to its higher affinity to non-polar adsorbates compared with the ordinary activated carbons [30]. Carbon content will be higher after physically activation [63]. Generally, the carbon content should increase with physical activation temperature, because there is an enrichment of elemental carbon, while the other elements, such as nitrogen, are largely removed with the off-gases during the physical activation [64]. Table 4 shows that the carbon content of both bioadsorbents increased as the ash content decreased significantly. This could have been caused by physical activation at high temperature in which most of the organic substances were degraded and discharged as gas and liquid tars, while leaving the material with high carbon purity [65,66]. As shown in table 4, CS and PKS bioadsorbent prepared under N_2_ activation have a higher carbon content compared with Air activation. Whereas, the ash content for both bioadsorbents produced under air activation was higher than under N_2_ physical activation. A greater value of ash content aids the limited combustible components kept in the pretreated biomass [67]. Under the circumstances, it was inadequate to carbonize the CS and PKS using only air as the carrier gas in the whole activation process.

**Table 4.** Contents of C, H, N, O and ash of the bioadsorbent (% oven-dried weight).

physical activation			CHNS and ash content											
			C		N		S		H		O		ash	
time (min)	gas carrier	temp. (°C)	CS	PKS	CS	PKS	CS	PKS	CS	PKS	CS	PKS	CS	PKS
control (non-treated bioadsorbent)														
raw material			45.93	46.12	0.94	0.79	0.01	0.03	10.79	5.50	42.33	47.56	0.17	0.20
75	N_2_	700	46.45	47.12	0.53	0.48	0.01	0.01	8.32	4.65	44.69	47.74	34.43	37.21
one-stage physical activation														
75	Air	700	63.03	59.22	0.38	0.39	0.01	0.01	8.15	4.56	28.43	35.82	9.95	9.65
75	N_2_	700	63.44	61.88	0.39	0.38	0.01	0.01	8.65	4.01	27.51	33.72	6.89	7.54
115	Air	700	54.23	49.76	0.42	0.43	0.01	0.01	9.12	5.07	36.22	44.73	13.65	14.11
115	N_2_	700	57.85	52.58	0.31	0.40	0.01	0.01	8.79	4.93	33.04	42.08	12.08	12.87
two-stage continuous physical activation														
115	Air > N_2_	700	—	62.03	—	0.35	—	0.06	—	1.40	—	36.16	6.13	9.28
115	Air > N_2_	800	56.90	—	0.07	—	0.06	—	6.76	—	36.21	—	8.13	14.37
115	N_2_ > Air	600	69.12	65.11	0.06	0.33	0.06	0.06	3.88	1.50	26.88	33.00	5.42	5.56

According to table 4, the carbon content of bioadsorbents prepared under one-stage physical activation is lower than two-stage continuous physical activation. The increased carbon content was due to the thermal degradation of volatile components [68]. The highest carbon content for CS and PKS bioadsorbent was 69.12 and 65.11% when both bioadsorbents were activated at 600°C under N_2_ > Air activation. Owing to the high carbon content, bioadsorbent which was prepared by two-stage continuous physical activation is the preferred adsorbent in comparison to one-stage physical activation. The increase of carbon content also indicates an increase in the polarity upon mild oxidation of N_2_ > Air activation. This indicates that the PKS and CS bioadsorbent fits into the active carbon. Moreover, the off-gases that develop during N_2_ > Air activation in an oxygen-poor environment are used for their energy potential. The remaining solid that is rich in elemental carbon may be used in various applications [64]. Table 4 illustrates that the ash content for bioadsorbent prepared under Air > N_2_ was higher than N_2_ > Air activation. The higher ash content from Air > N_2_ activation suggests the presence of higher amount of oxides or inorganic impurities formed at the surface of bioadsorbent. It is known that the presence of ash can affect the chemical characteristics and the adsorptive behaviour of activated carbon [68]. Thus, bioadsorbent with the low level of ash obtained via N_2_ > Air activation obtained in the present study has a desirable characteristic. The elemental analysis revealed that the sample with the highest carbon content was prepared via N_2_ > Air activation, which supports the fact that this physical activation is suitable for producing high-grade activated carbon. Nitrogen content and sulfur content of the samples prepared in two-stage continuous physical activation were very low in the analysis. For elemental analysis, oxygen content is also an important aspect, because it can form surface oxygen functional groups, such as carboxylic acid and carbonyls that have an impact on the adsorption process [55,69]. The higher the oxygen content, the lower the amount of surfactant adsorbed [70]. In this present study, N_2_ > Air activation formed highly porous bioadsorbent with oxygen content that is lower than other carbon materials produced by conventional processes.

### 3.6. FTIR

Evaluations of the surface chemical characteristics in this study were performed only on the bioadsorbent that obtained the highest adsorption properties and the highest surface area for both bioadsorbent prepared under one-stage physical activation (N_2_ for at 700°C) and two-stage continuous physical activation (N_2_ > Air at 600°C).

**Figure 6.**
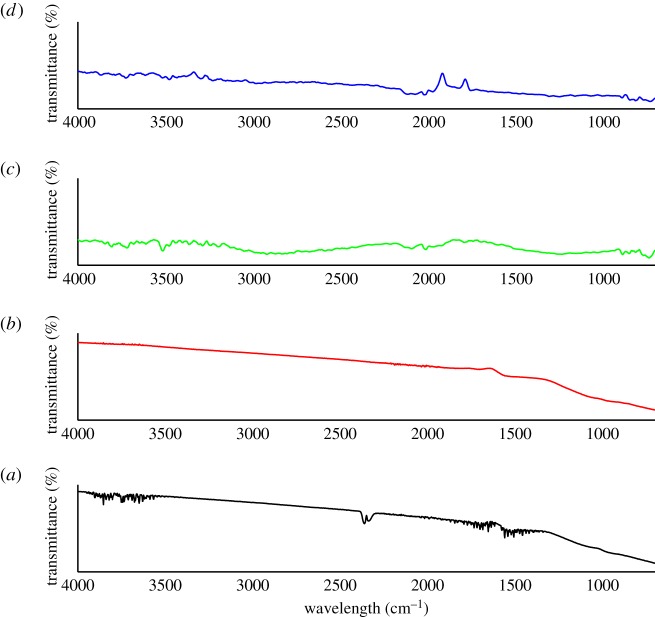
FTIR spectra of bioadsorbent derived from CS and PKS prepared under one-stage and two-stage continuous physical activation where (*a*,*b*) CS and PKS bioadsorbent prepared under N_2_ activation at 700°C, respectively, and (*c*,*d*) CS and PKS bioadsorbent under N_2_ > Air activation with temperature at 600°C.

Figure 6 displays the FTIR spectra of bioadsorbents derived from CS and PKS prepared under N_2_ single physical activation and N_2_ > Air two-stage continuous physical activation. Figure 6 suggests that N-containing groups were introduced to the structure of bioadsorbent that was prepared under N_2_ activation. Peaks of 1539, 1558 and 1618 cm^−1^; and 1558 and 3319 cm^−1^ indicate the presence of N–H stretch on the CS and PKS bioadsorbent, respectively [71]. The higher adsorption rate and capacity toward heavy metal was obtained in the case of nitrogenated carbons. In this aspect, nitrogen-containing porous carbons with high surface area are recently receiving a great deal of attention due to their specific structures and unique properties [72–75]. For example, they show enhanced adsorption capacity of anions, resulting from a positively charged carbon surface, and improved thermal stability by incorporating heteroatoms when they are used as catalytic supports [75].

The new appearance of bands from 650 to 900 cm^−1^ indicate an increase in aromatic nature for the activated compounds [76] prepared under N_2_ > Air activation. The peaks of 742, 812 and 899 cm^−1^ in CS bioadsorbent and 723, 781, 814 and 916 cm^−1^ might be due to the presence of C–H out of plane bending in benzene derivatives. FTIR reveals the broad peaks at 1213 and 1212 cm^−1^ on CS and PKS bioadsorbents under two-stage continuous activation, respectively. Peaks detected at 1200–1300 cm^−1^ attribute to C–O–C group of stretching mode in acids, alcohols, phenols, ethers and esters [77,78]. It can be stated that N_2_ > Air activation formed ester linkages with the –OH groups helping to cross-link the polymer chains of the bioadsorbent. Additionally, the general increase in band intensities with activation treatment from around 600 to 1300 cm^−1^ is indicative of phosphorus-containing groups being formed during thermal activation [76]. Two-stage continuous physical activation (N_2_ > Air) also promoted two sharp peaks at 1368 and 1578 cm^−1^ for CS bioadsorbent and one sharp peak at 1368 cm^−1^ for PKS bioadsorbent. These sharp peaks can be ascribed to the asymmetric COO^−^ vibration and symmetric COO^−^ vibration, respectively [79,80]. Absorptions at 1578 cm^−1^ present on CS adsorbent correspond to C–C stretching vibrations and NH_2_ in the benzene ring. Based on the study, both bioadsorbents expressed a more distinct absorbance by oxygenated groups at 1740 cm^−1^. This peak absorption reveals that mild oxidation from N_2_ > Air physical activation increases the concentration of carbonyl functional groups on the surface of the CS and PKS bioadsorbent. Mild air oxidation generates a more uniform spatial distribution of oxygenated functional groups for the adsorbent [81]. In addition, bands at 1725–1740 cm^−1^ are the characteristic stretching vibration of carbonyl group C=O in esters and carboxylic acids [79,80]. Whereas, there are strong peaks located at 2302 and 2384 cm^−1^ for CS bioadsorbent and 2302 and 2346 cm^−1^ in PKS bioadsorbent. These peaks assign to the vibration of C≡N and C≡C, respectively [82]. CS bioadsorbent showed a weak but visible absorption at 2564 cm^−1^ considered as S–H bond stretching vibration. These clearly demonstrated that –SH group had been successfully grafted onto the surface of CS bioadsorbent prepared under N_2_ > Air physical activation. Figure 6 displays the peaks of 2967 cm^−1^ in CS bioadsorbent and 2869 and 2972 cm^−1^ in PKS bioadsorbent which represent the characteristic of C–H stretch of imidazole. Anisuzzaman [77] stated that spectra range between 2800 and 3000 cm^−1^ refers to the C–H stretching in the alkane group. Likewise, figure 6 indicates spectra's vibrations, implied that it may have the presence of monomeric alcohols or phenols due to the wavenumbers corresponding to 3010, 3439 and 3670 cm^−1^ for CS bioadsorbent and 3635 cm^−1^ for PKS bioadsorbent prepared under N_2_ > Air activation. Anisuzzaman [77] also reported that the peaks detected in the spectra at bandwidths of 3300–3800 cm^−1^ represent O–H stretching vibration of surface hydroxylic functional groups in low concentrations. Besides, the band of O–H stretching vibrations reveals the existence of chemisorbed water from the activated bioadsorbent. The asymmetry of this band indicates the presence of strong hydrogen bonds in the adsorbent [83].

The surface chemistry and pore structure of porous carbons determine its application. The surface chemistry could be modified by various methods, such as acid treatment, oxidization, ammonization, plasma, microwave treatment and others. Oxygen-containing functional groups have been devoted as the main groups responsible for heavy metal binding on the activated carbon surface [48]. It was found that the N_2_ > Air two-stage continuous physical activation not only greatly increased the specific surface area but also caused the formation of oxygen-containing groups (−OH and C=O) on the CS and PKS bioadsorbent surface. Oxidation is one of the most conventional modification techniques used to induce or enhance oxygen functional groups on the surface of activated carbon such as carboxylic, lactones, phenols, ketones, quinones, hydroxyl and carboxylic anhydride [48,84]; it can also remove the mineral elements and improve the hydrophilic nature of the surface [48,85]. These acidic surface groups are polar and enhance the ion exchange properties of the carbon, thereby increasing the adsorption of cation [48,85]. Thus, the surface oxygen-containing functional group had been adequately introduced to the bioadsorbent via N_2_ > Air activation in this study. Owing to the high surface areas and well-developed porous structures, and the presence of different types of functional groups on their surfaces, the porous bioadsorbent prepared via N_2_ > Air may be applied in many areas including the protection of natural environment by removing sulfur compounds, nitrogen oxides and carbon dioxide; purification of wastewater by removing heavy metal ions, aromatic compounds and dye molecules; use of catalysis or catalyst supports and others.

### 3.7. TGA

Evaluations of the thermal behaviour characteristics in this study were performed only on the bioadsorbent that obtained the highest adsorption properties and the highest surface area for both bioadsorbents prepared under N_2_ > Air two-stage continuous physical activation at 600°C.

**Figure 7.**
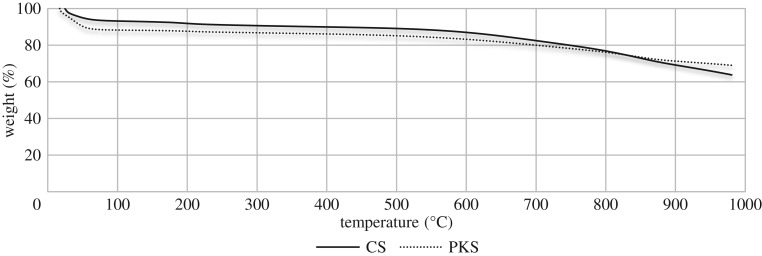
TGA of bioadsorbent derived from CS and PKS prepared under two-stage continuous physical activation.

By analysing the TGA results, we found that bioadsorbent prepared under two-stage continuous activation gained a low weight loss. Three zones of the profile are obtained for both cases. Apparently with respect in increasing temperature from 400 to 800°C, the total weight loss for pretreated CS and PKS are 22.29 and 16.36%, respectively. From this result (figure 7), it was found that both physically activated bioadsorbents exhibit high thermal stability. The high thermal stability behaviour may be due to the new oxygen functional groups introduced on the bioadsorbent via two-stage continuous activation. Some researchers had stated oxygen-containing function groups, such as anhydride, lactones and epoxy groups containing –C–O–C–, exhibited better thermal stability and were only decomposed at a higher temperature [86,87]. Approximately 63.83 and 69.01% of the mass for CS and PKS bioadsorbent, respectively, are still not volatilized at 1000°C. The greater thermal stability of the activated bioadsorbents is also thought to be because they have more stable structures with higher carbon assay [88]. Thus, TGA result displays that both bioadsorbents prepared via N_2_ > Air activation achieved high amount of residue.

## 4. Conclusion

Ash leaching method with phosphoric acid prior to physical activation is able to remove a huge fraction of ash-forming elements in biomass; however, it is unable to remove ash components such as silica or silicate from high-ash agricultural biomass such as PKS and CS. Removal of the silica compounds attached to the surface of the agriculture biomass opens up more reactive surfaces for the physical activation process and, thus, creates a bioadsorbent with a higher surface area. From this study, higher reduction of silica was achieved by additional stage of air activation after N_2_ activation (N_2_ > Air) which increased the development of pores structure on the bioadsorbent. This novel process was able to expose mesopores and micropores that were previously covered/clogged in nature, and simultaneously create new pores. The optimum condition to achieve the highest BET value for H_3_PO_4_ pretreated PKS and CS is via two-stage continuous physical activation of N_2_ > Air under 600°C activation temperature. Introducing low-temperature activation process as applied in this study modestly increases the feasible expansion potential towards industrial scale. This novel process of two-stage continuous physical activation meets the recent industrial adsorbent requirements of low activation temperature, high fixed carbon content, high yield, high adsorption properties and high surface area, which are the key factors in large-scale production and usage.

## Data accessibility

Adsorption properties; BET surface area; N_2_ adsorption; FTIR and TGA files have been uploaded to the Dryad Digital Repository at: http://dx.doi.org/10.5061/dryad.ch8gf53 [89].

## References

[1] Kundu A, Gupta BS, Hashim MA, Sahu JN, Mujawar M, Redzwan G 2015 Optimisation of the process variables in production of activated carbon by microwave heating. RSC Adv. 5, 35 899– 35 908. (10.1039/C4RA16900J)

[2] Abood MM, Rajendiran J, Azhari NN 2015 Agricultural waste as low cost adsorbent for the removal of Fe (II) ions from aqueous solution. Infrastruct. Univ. Kuala Lumpur Res. J. 3, 29–39.

[3] Abdel-Ghani NT, Hefny M, El-Chaghaby GAF 2007 Removal of lead from aqueous solution using low cost abundantly available adsorbents. Int. J. Environ. Sci. Technol. 4, 67–73. (10.1007/BF03325963)

[4] Rahman MM, Adil M, Yusof AM, Kamaruzzaman YB, Ansary RH 2014 Removal of heavy ions with acid activated carbons derived from oil palm and coconut shell. Material 7, 3634–3650. (10.3390/ma7053634) PMC5453229 28788640

[5] Ioannidou O, Zabaniotou A 2007 Agricultural residues as precursors for activated carbon production—a review. Renew. Sustain. Energy Rev. 11, 1966–2005. (10.1016/j.rser.2006.03.013)

[6] Olivares-Marín M, Fernández-González C, MacÍas-García A, Gómez-Serrano V 2012 Preparation of activated carbon from cherry stones by physical activation in air. Influence of the chemical carbonisation with H_2_SO_4_. J. Anal. Appl. Pyrolysis 94, 131–137. (10.1016/j.jaap.2011.11.019)

[7] Hong J, Xiaomin W, Zhengrong G 2013 Hierarchical carbon materials from high ash bio-char of distiller's dried grain with solubles for supercapacitor. Mater. Focus 2, 105–112. (10.1166/mat.2013.1059)

[8] Brewer CE, Unger R, Schmidt-Rohr K, Brown RC 2011 Criteria to select biochars for field studies based on biochar chemical properties. Bioenergy Res. 4, 312–323. (10.1007/s12155-011-9133-7)

[9] Molina-Sabio M, Rodríguez-Reinoso F 2004 Role of chemical activation in the development of carbon porosity. Colloids Surfaces A Physicochem. Eng. Asp. 241, 15–25. (10.1016/j.colsurfa.2004.04.007)

[10] Nahil MA, Williams PT 2012 Characterisation of activated carbons with high surface area and variable porosity produced from agricultural cotton waste by chemical activation and co-activation. Waste Biomass Valorization 3, 117–130. (10.1007/s12649-012-9109-7)

[11] Xiao F, Pignatello JJ 2016 Effects of post-pyrolysis air oxidation of biomass chars on adsorption of neutral and ionizable compounds. Environ. Sci. Technol. 50, 6276–6283. (10.1021/acs.est.6b00362) 27152745

[12] Ahmadpour A, Do DD 1996 The preparation of active carbons from coal by chemical and physical activation. Carbon NY 34, 471–479. (10.1016/0008-6223(95)00204-9)

[13] Ahmadpour A, Do DD 1997 The preparation of activated carbon from macadamia nutshell by chemical activation. Carbon NY 35, 1723–1732. (10.1016/S0008-6223(97)00127-9)

[14] Caturla F, Molina-Sabio M, Rodríguez-Reinoso F 1991 Preparation of activated carbon by chemical activation with ZnCl_2_. Carbon NY 29, 999–1007. (10.1016/0008-6223(91)90179-M)

[15] Hu Z, Srinivasan M 1999 Preparation of high-surface-area activated carbon from coconut shell. Microporous Mesoporous Mater. 27, 11–18. (10.1016/j.carbon.2010.03.059)

[16] Laine J, Calafat A 1989 Preparation and characterization of activated carbon from coconut shell impregnated with phosphoric acid. Carbon NY 27, 191–195. (10.1016/0008-6223(89)90123-1)

[17] Lua AC, Yang T 2005 Characteristics of activated carbon prepared from pistachio-nut shell by zinc chloride activation under nitrogen and vacuum conditions. Colloid Interface Sci. 290, 505–513. (10.1016/j.jcis.2005.04.063) 16002081

[18] Molina-Sabio M, Rodríguez-Reinoso F, Caturla F, Sellés MJ 1996 Development of porosity in combined phosphoric acid-carbon dioxide activation. Carbon NY 34, 457–462. (10.1016/0008-6223(95)00209-X)

[19] Otowa T, Nojima Y, Miyazaki T 1997 Development of KOH activated high surface area carbon and its application to drinking water purification. Carbon NY 35, 1315–1319. (10.1016/S0008-6223(97)00076-6)

[20] Rodríguez-Reinoso F, Molina-Sabio M 1992 Activated carbons from lignocellulosic materials by chemical and/or physical activation: an overview. Carbon NY 30, 1111–1118. (10.1016/0008-6223(92)90143-K)

[21] Rwayhah YM, Hassan M, Shehata M 2017 Nanoporous activated carbon from olive stones wastes. J. Sci. Ind. Res. 76, 725–732.

[22] Kohls DJ, Beaucage G 2002 Rational design of reinforced rubber. Solid State Mater. Sci. 6, 183–194. (10.1016/S1359-0286(02)00073-6)

[23] Vernersson T, Bonelli PR, Cerrela EG, Cukierman AL 2002 *Arundo donax* cane as a precursor for activated carbons preparation by phosphoric acid activation. Bioresour. Technol. 83, 95–104. (10.1016/S0960-8524(01)00205-X) 12056497

[24] Mahinpey N, Murugan P, Mani T, Raina R 2009 Analysis of bio-oil, biogas, and biochar from pressurized pyrolysis of wheat straw using a tubular reactor. Energy Fuels 23, 2736–2742. (10.1021/ef8010959)

[25] Hung JJ 2012 The production of activated carbon from coconut shells using pyrolysis and fluidized bed reactors. University of Arizona See http://arizona.openrepository.com/arizona/bitstream/10150/243968/1/azu_etd_mr_2012_0079_sip1_m.pdf.

[26] Lee CL, H'ng PS, Paridah MT, Chin KL, Khoo PS, Nazrin RAR, Asyikin SN, Mariusz M 2017 Effect of reaction time and temperature on the properties of carbon black made from palm kernel and coconut shell. Asian J. Sci. Res. 10, 24–33. (10.3923/ajsr.2017.Research)

[27] Anonymous. 1992 *Japanese Standard Association. Standard testing method of methylene blue number of activated carbon 1470–1991*.

[28] Joshi S, Pokharel BP 2014 Preparation and characterization of activated carbon from Lapsi (*Choerospondias axillaris*) seed stone by chemical activation with potassium hydroxide. J. Inst. Eng. 9, 79–88. (10.3126/jie.v9i1.10673)

[29] Chin KL, H'ng PS, Go WZ, Wong WZ, Lim TW, Maminski M, Paridah MT, Luqman AC 2013 Optimization of torrefaction conditions for high energy density solid biofuel from oil palm biomass and fast growing species available in Malaysia. Ind. Crops Prod. 49, 768–774. (10.1016/j.indcrop.2013.06.007)

[30] Patnukao P, Pavasant P 2008 Activated carbon from *Eucalyptus camaldulensis Dehn* bark using phosphoric acid activation. Bioresour. Technol. 99, 8540–8543. (10.1016/j.biortech.2006.10.049) 18455392

[31] Asadullah M, Kabir MS, Ahmed MB, Razak NA, Rasid NSA, Aezzira A 2013 Role of microporosity and surface functionality of activated carbon in methylene blue dye removal from water. Korean J. Chem. Eng. 30, 2228–2234. (10.1007/s11814-013-0172-y)

[32] Liu X, He C, Yu X, Bai Y, Ye L, Wang B, Zhang L 2018 Net-like porous activated carbon materials from shrimp shell by solution-processed carbonization and H_3_PO_4_ activation for methylene blue adsorption. Powder Technol. 326, 181–189. (10.1016/j.powtec.2017.12.034)

[33] Novais RM, Ascensão G, Tobaldi DM, Seabra MP, Labrincha JA 2018 Biomass fly ash geopolymer monoliths for effective methylene blue removal from wastewaters. J. Clean Prod. 171, 783–794. (10.1016/j.jclepro.2017.10.078)

[34] Babatunde OA, Garba S, Ali ZN 2016 Surface modification of activated carbon for improved iodine and carbon tetrachloride adsorption. Am. J. Chem. 6, 74–79. (10.5923/j.chemistry.20160603.02)

[35] Itodo A, Abdulrahman F, Hassan L, Maigandi S, Itodo H 2010 Application of methylene blue and iodine adsorption in the measurement of specific surface area by four acid and salt treated activated carbons. New York Sci. J. 3, 25–33.

[36] Kyritsis S 2001 1st world conference on biomass for energy and industry. II. Sevilla, Spain: James & James (Science Publishers) Ltd.

[37] Montes V, Hill JM 2018 Pore enlargement of carbonaceous materials by metal oxide catalysts in the presence of steam: influence of metal oxide size and porosity of starting material. Microporous Mesoporous Mater. 256, 91–101. (10.1016/j.micromeso.2017.08.001)

[38] Costa L, Avataneo M, Bracco P, Brunella V 2002 Char formation in polyvinyl polymers I. Polyvinyl acetate. Polym. Degrad. Stab. 77, 503–510. (10.1016/S0141-3910(02)00108-8)

[39] Cuhadar C 2005 Production and characterization of activated carbon from hazelnut shell and hazelnut husk. Ankara, Turkey: Middle East Technical University.

[40] Lee SY, Park SJ 2013 Determination of the optimal pore size for improved CO_2_ adsorption in activated carbon fibers. J. Colloid Interface Sci. 389, 230–235. (10.1016/j.jcis.2012.09.018) 23046640

[41] Huang PH, Cheng HH, Lin SH 2015 Adsorption of carbon dioxide onto activated carbon prepared from coconut shells. J. Chem. 2015, 1–10.

[42] Hutchins RA 1973 Economic factors in granular carbon thermal regeneration. Chem. Eng. Prog. 69, 48–55.

[43] Hu X, Radosz M, Cychosz KA, Thommes M 2011 CO_2_-filling capacity and selectivity of carbon nanopores: synthesis, texture, and pore-size distribution from quenched-solid density functional theory (QSDFT). Environ. Sci. Technol. 45, 7068–7074. (10.1021/es200782s) 21721529

[44] Gottipati R 2012 Preparation and characterization of microporous activated carbon from biomass and its application in the removal of chromium (VI) from aqueous phase. Odisha, India: National Institute of Technology Rourkela.

[45] Olawale AS, Ajayi OA 2009 Thermal activation of *Canarium Schweinfhurthi* nutshell. Aust. J. Basic Appl. Sci. 3, 3801–3807.

[46] Bikshapathi M, Verma N, Singh RK, Joshi HC, Srivastava A 2011 Preparation of activated carbon fibers from cost effective commercial textile grade acrylic fibers. Carbon Lett. 12, 44–47. (10.5714/CL.2011.12.1.044)

[47] Gratuito MKB, Panyathanmaporn T, Chumnanklang R 2008 Production of activated carbon from coconut shell: optimization using response surface methodology. Bioresour. Technol. 99, 4887–4895. (10.1016/j.biortech.2007.09.042) 17993271

[48] Bohli T, Ouederni A 2016 Improvement of oxygen-containing functional groups on olive stones activated carbon by ozone and nitric acid for heavy metals removal from aqueous phase. Environ. Sci. Pollut. Res. 23, 15 852–15 861. (10.1007/s11356-015-4330-0) 25794582

[49] Shafeeyan MS, Daud WM, Houshmand A, Arami-Niya A 2011 Ammonia modification of activated carbon to enhance carbon dioxide adsorption: effect of pre-oxidation. Appl. Surf. Sci. 257, 3936–3942. (10.1016/j.apsusc.2010.11.127)

[50] Jansen RJJ, van Bekkum H 1994 Amination and ammoxidation of activated carbons. Carbon NY 32, 1507–1516. (10.1016/0008-6223(94)90146-5)

[51] Stöhr B, Boehm HP, Schlögl R 1991 Enhancement of the catalytic activity of activated carbons in oxidation reactions by thermal treatment with ammonia or hydrogen cyanide and observation of a superoxide species as a possible intermediate. Carbon NY 29, 707–720. (10.1016/0008-6223(91)90006-5)

[52] Abechi SE, Gimba CE, Uzairu A, Dallatu YA 2013 Preparation and characterization of activated carbon from palm kernel shell by chemical activation. Res. J. Chem. Sci. 3, 54–61.

[53] Wafti NSA, Harrison LLN, Loh SK, Astimar AA, Zulkifli AR, Choo YM 2017 Activated carbon from oil palm biomass as potential adsorbent for palm oil mill effluent treatment. J. Oil Palm Res. 29, 278–290. (10.21894/jopr.2017.2902.12)

[54] De Gisi S, Lofrano G, Grassi M, Notarnicola M 2016 Characteristics and adsorption capacities of low-cost sorbents for wastewater treatment: a review. Sustain. Mater. Technol. 9, 10–40. (10.1016/j.susmat.2016.06.002)

[55] Guan BTH, Latif PA, Yap TY 2013 Physical preparation of activated carbon from sugarcane bagasse and corn husk and its physical and chemical characteristics. Int. J. Eng. Res. Sci. Technol. 2, 1–14.

[56] Bouchelta C, Salah Medjram M, Bertrand O, Bellat JP 2008 Preparation and characterization of activated carbon from date stones by physical activation with steam. J. Anal. Appl. Pyrolysis 82, 70–77. (10.1016/j.jaap.2007.12.009)

[57] Smíšek M, Černý S 1970 Active carbon: manufacture, properties and applications. New York, NY: Elsevier Publishing Company.

[58] El-Hendawy AN, Alexander AJ, Andrews RJ, Forrest G 2008 Effects of activation schemes on porous, surface and thermal properties of activated carbons prepared from cotton stalks. J. Anal. Appl. Pyrolysis 82, 272–278. (10.1016/j.jaap.2008.04.006)

[59] Jung S, Oh S, Choi G, Kim J 2014 Production and characterization of microporous activated carbons and metallurgical bio-coke from waste shell biomass. J. Anal. Appl. Pyrolysis 109, 123–131. (10.1016/j.jaap.2014.07.003)

[60] Savova D, Apak E, Ekinci E, Yardim F, Petrov N, Budinova T, Razvigorova M, Minkova V 2001 Biomass conversion to carbon adsorbents and gas. Biomass Bioenergy 21, 133–142. (10.1016/S0961-9534(01)00027-7)

[61] Razali WAW, Baharuddin AS, Tarmezee Talib A, Sulaiman A, Naim MN, Hassan MA, Shirai Y 2012 Degradation of oil palm empty fruit bunches (OPEFB) fibre during composting process using in-vessel composter. BioResources 7, 4786–4805. (10.15376/biores.7.4.4786-4805)

[62] del Campo BG 2015 Production of activated carbon from fast pyrolysis biochar and the detoxification of pyrolytic sugars for ethanol fermentation. Ames, IA: Iowa State University.

[63] Sylvia N, Hakim L, Fardian N, Yunardi 2018 Adsorption performance of fixed-bed column for the removal of Fe (II) in groundwater using activated carbon made from palm kernel shells. In 3rd Int. Conf. on Chemical Engineering Sciences and Applications 2017 (3rd ICChESA 2017), 20–21 September, Banda Aceh, Indonesia, pp. 1–9. IOP Publishing Ltd (10.1088/1757-899X/334/1/012030)

[64] Shinogi Y, Kanri Y 2003 Pyrolysis of plant, animal and human waste: physical and chemical characterization of the pyrolytic products. Bioresour. Technol. 90, 241–247. (10.1016/S0960-8524(03)00147-0) 14575946

[65] Tan IA, Ahmad AL, Hameed BH 2008 Preparation of activated carbon from coconut husk: optimization study on removal of 2, 4, 6-trichlorophenol using response surface methodology. J. Hazard. Mater. 153, 709–717. (10.1016/j.jhazmat.2007.09.014) 17935879

[66] Supriya S, Palanisamy PN, Shanthi P 2014 Preparation and characterization of activated carbon from Casuarina for the removal of dyes from textile wastewater. Int. J. ChemTech. Res. 6, 3635–3641.

[67] Lu K, Lee W, Chen W, Liu S, Lin T 2012 Torrefaction and low temperature carbonization of oil palm fiber and eucalyptus in nitrogen and air atmospheres. Bioresour. Technol. 123, 98–105. (10.1016/j.biortech.2012.07.096) 22940305

[68] Salgado MDF, Abioye AM, Junoh MM, Santos JAP, Ani FN 2018 Preparation of activated carbon from babassu endocarpunder microwave radiation by physical activation. IOP Conf. Ser. Earth Environ. Sci. 105, 1–13. (10.1088/1755-1315/105/1/012116)

[69] Wu SH, Pendleton P 2001 Adsorption of anionic surfactant by activated carbon: effect of surface chemistry, ionic strength, and hydrophobicity. J. Colloid Interface Sci. 243, 306–315. (10.1006/jcis.2001.7905)

[70] Pendleton P, Wu SH, Badalyan A 2002 Activated carbon oxygen content influence on water and surfactant adsorption. J. Colloid Interface Sci. 246, 235–240. (10.1006/jcis.2001.8052) 16290407

[71] Coates J 2006 Interpretation of infrared spectra, a practical approach. Encycl. Anal. Chem. 12, 10 815–10 837. (10.1002/9780470027318.a5606)

[72] Delgado JL, Bouit PA, Filippone S, Herranz MÁ, Martín N 2010 Organic photovoltaics: a chemical approach. Chem. Commun. 46, 4853–4865. (10.1039/c003088k) 20498918

[73] Meier MS, Andrews R, Jacques D, Cassity KB, Qian D 2008 Tearing open nitrogen-doped multiwalled carbon nanotubes. J. Mater. Chem. 18, 4143–4145. (10.1039/b809348b)

[74] Ozaki JI, Tanifuji SI, Kimura N, Furuichi A, Oya A 2006 Enhancement of oxygen reduction activity by carbonization of furan resin in the presence of phthalocyanines. Carbon NY 44, 1324–1326. (10.1016/j.carbon.2005.12.026)

[75] Shen W, Fan W 2013 Nitrogen-containing porous carbons: synthesis and application. J. Mater. Chem. A 1, 999–1013. (10.1039/c2ta00028h)

[76] Klasson KT, Wartelle LH, Rodgers JE, Lima IM 2009 Copper(II) adsorption by activated carbons from pecan shells: effect of oxygen level during activation. Ind. Crops Prod. 30, 72–77. (10.1016/j.indcrop.2009.01.007)

[77] Anisuzzaman SM, Joseph CG, Taufiq-Yap YH, Krishnaiah D, Tay VV 2015 Modification of commercial activated carbon for the removal of 2,4-dichlorophenol from simulated wastewater. J. King Saud. Univ. Sci. 27, 318–330. (10.1016/j.jksus.2015.01.002)

[78] Foo KY, Hameed BH 2009 Utilization of biodiesel waste as a renewable resource for activated carbon: application to environmental problems. Renew. Sustain. Energy Rev. 13, 2495–2504. (10.1016/j.rser.2009.06.009)

[79] Jia Y. F., Thomas KM 2000 Adsorption of cadmium ions on oxygen surface sites in activated carbon. Langmuir 16, 1114–1122. (10.1021/la990436w)

[80] Deng L, Lu B, Li J, Lv G, Du S, Shi J, Yang Y 2017 Effect of pore structure and oxygen-containing groups on adsorption of dibenzothiophene over activated carbon. Fuel 200, 54–61. (10.1016/j.fuel.2017.03.018)

[81] Bardestani R, Kaliaguine S 2018 Steam activation and mild air oxidation of vacuum pyrolysis biochar. Biomass Bioenergy 108, 101–112. (10.1016/j.biombioe.2017.10.011)

[82] Li M, Yu C, Hu C, Zhao C, Zhang M, Ding Y, Wang X, Qiu J 2018 Template-free synthesis of interconnected carbon nanosheets via cross-linking coupled with annealing for high-efficiency triiodide reduction. RSC Adv. 20, 250–254. (10.1039/C7GC02701 J)

[83] Biniak S, Szymański G, Siedlewski J, Świątkowski A 1997 The characterization of activated carbons with oxygen and nitrogen surface groups. Carbon NY 35, 1799–1810. (10.1016/S0008-6223(97)00096-1)

[84] Bansal RC, Goyal M 2005 Activated carbon adsorption. Boca Raton, FL: CRC Press.

[85] Shen W, Li Z, Liu Y 2008 Surface chemical functional groups modification of porous carbon. Recent Patents Chem. Eng. 1, 27–40. (10.2174/2211334710801010027)

[86] Figueiredo JL, Pereira MFR 2010 The role of surface chemistry in catalysis with carbons. Catal. Today 150, 2–7. (10.1016/j.cattod.2009.04.010)

[87] Hotova G, Slovak V, Soares OSGP, Figueiredo JL, Pereira MFR 2018 Oxygen surface groups analysis of carbonaceous samples pyrolysed at low temperature. Carbon NY 134, 255–263. (10.1016/j.carbon.2018.03.067)

[88] Kim J-M, Song I-S, Cho D, Hong I 2011 Effect of carbonization temperature and chemical pre-treatment on the thermal change and fiber morphology of kenaf-based carbon fibers. Carbon Lett. 12, 131–137. (10.5714/CL.2011.12.3.131)

[89] Lee et al 2018 Data from: Production of bioadsorbent from phosphoric acid pretreated palm kernel shell and coconut shell by two-stage continuous physical activation via N_2_ and air *Dryad Digital Repository*. (10.5061/dryad.ch8gf53) PMC6304154 30662718

